# Analysis of factors associated with dropping-out from HIV antiretroviral therapy in Kunming City, China

**DOI:** 10.1186/s12879-019-4658-z

**Published:** 2019-12-10

**Authors:** Bin Liao, Xiao-Wen Zhang, Jing-Ying Wang, Jun Liu, Jun Liang, Wen-Jie He, Biao Hong, Yi Li

**Affiliations:** 1 Department of AIDS and Sexually Transmitted Disease Control and Prevention, Kunming Center for Disease Control and Prevention, Kunming, Yunnan China; 2 Department of AIDS Control and Prevention, Panlong District for Disease Control and Prevention, Kunming, Yunnan China

**Keywords:** HIV infection, Antiretroviral therapy, Drop out

## Abstract

**Background:**

The implementation of national antiretroviral therapy (ART) and expanded ART policies results in that more and more HIV-infected patients receive ART in Kunming, Yunnan province, China. At the same time, however, the number of patients, who drop-out from ART, are also increasing. In this study, we explored the factors that may account for drop-out.

**Methods:**

Four hundred and thirty-nine HIV-infected patients, who received or used to receive ART, were recruited in this study. Their age is among 18 and 75. All patients were divided into two group: ART group (187 patients) and drop-out group (252 patients). Appropriate bio-statistics analysis, including univariate analysis and Multivariate analysis, were used to identify factors associated with drop-out.

**Results:**

Data from all patients were analyzed. Univariate analysis suggested that the factors associated with drop-out may include age, residential area, educational level, occupation, monthly income, the access to minimum living allowance, HIV transmission route, and living status. On the other hand, factors including area, monthly income, the access to minimum living allowance, and referral methods of follow-up institutions account for drop-out in multivariate analysis.

**Conclusions:**

This study identified a number of factors associated with drop out from ART. Based on our findings,appropriate interventions should be introduced decrease drop-out.

## Background

Kunming is the capital of Yunnan Province. It is in the southwest of China. The number of population is about 6.8 million. Kunming city is the political, economic and cultural center of Yunnan Province. In 1991, the first local HIV-infected case was reported in Kunming. By the end of 2018, there are about 15,000 infected patients living with HIV or AIDS. Kunming makes up 14% HIV-patients of Yunnan province. Antiretroviral therapy (ART) is the key and effective strategy for AIDS prevention and control. ART can prolong life for HIV-infected patients [1], and prevent continued HIV transmission [2]. To expand the coverage rate of ART, achieve therapy success are the common goals of the country, health-care doctors and HIV infected patients [3]. The world is embarking on a Fast-Track strategy to end the AIDS epidemic by 2030 [4]. Since the implementation of the national free antiretroviral therapy and expanding therapy policies, the number of HIV infected people receiving ART is increasing [5, 6], the number of people dropping out from ART also increasing year by year [6, 7]. Controlling the dropout rate of antiretroviral therapy can effectively reduce the mortality rate of AIDS, contribute to the implementation of the fast track strategy and realization of three 90% goals. The purpose of this study is to analyze the reasons and influencing factors of HIV-infected people dropping out from antiretroviral therapy in Kunming city, and explore the measures to reduce the rate of dropout from ART.

## Methods

### Study design and objective

The HIV epidemic situation data and antiretroviral therapy data were derived from the National AIDS Integrated Prevention and Control Information System. The data was taken from January 1, 1997 to August 31, 2018. HIV-infected people receiving antiretroviral therapy in the database were selected as the group retained in care, those who had received antiretroviral therapy but are currently dropped out as the drop out group. The types of drop out in our study includes drop-out from follow-up and stop taking ART medicine.

Kunming Center for Disease Control and Prevention (KMCDC) has designed and conducted a questionnaire. At first, we conducted a pre-investigation by qualitative interviews. According to the results we modified before finalizing questionnaire. The contents of the questionnaire include demographic characteristics, HIV/AIDS infection situation, services related to HIV antiretroviral therapy, etc.

Using the method of convenient sampling, the study included HIV-infected patients from 14 counties of Kunming City. All respondents including retained and drop out patients could contacted by CDC staff. Before the field interviews, we conducted a unified training for investigators of KMCDC. With the consent of the respondents by verbal,one-to-one interviews have been conducted. Drop out group and group retained in care use two sets of questionnaires. In our study, strict quality control was carried out in each stages of project design, data collection and data analysis.

### Definitions

Drop-out from follow-up refers after the appointed follow-up time more than 90 days, the patients did not actively contact the follow-up doctors or doctors could not contact the patient, and did not know the reasons for the discontinuation of therapy. Discontinuation refers to patients’ discontinuation of therapy for toxic side effects or various reasons, even doctors can contact patients, but patients no longer want to receive therapy.

### Inclusion criteria

According to the definition, we use inclusion criteria to select the participants to control the selection bias.

Inclusion criteria of the drop out group were: (1) age 18–75; (2) those who dropped out from follow-up or stopped taking ART medicine after therapy before our study; (3) without serious diseases or mental disorders.

Inclusion criteria of the group retained in care were: (1) age 18–75; (2) retained in antiretroviral therapy; (3) without serious diseases or mental disorders.

### Statistical analysis

When the questionnaire data entered in computer, verification files set to reduce the input errors. Input data twice to check consistency. Correct the discovered errors in time. STATA version 11.0 was used for analysis data. Univariate analysis was performed by Chi-square test, *P* value 0.05 was considered statistically significant.

Then the meaningful factors in single factor analysis were put into multivariate analysis. Logistic regression was used to analyze the influencing factors of the drop-out from antiretroviral therapy. The level of significance was defined as *P* < 0.05.

## Results

A total of 439 valid questionnaires were collected. 252 cases were in the drop out group and 187 cases were in the group retained in care.

### Social demographic characteristics and general situations of drop out group

There were 189 males (75%) and 63 females (25%) with average age of 39.46 ± 11.88 years. There were 215 cases of Han nationality (85.3%). The marital status was mainly single or divorced, with 174 cases (69.0%). 176 cases (69.8%) educational level were junior high school or below. Their main occupations were farmers, housekeeping and unemployment accounting for 70.2% totally.84 cases (33.3%) had monthly income less than 500 CNY. 190 cases (75.4%) didn’t have minimum living allowances. The HIV transmission route:133 cases (52.8%) were heterosexual transmission,43 cases (17.1%) were homosexual transmission, and 76 cases (30.1%) were injection drug abuse transmission. By living status,111 cases (44.0%) were living alone, 94 cases (37.3%) were living with their families.

### Univariate analysis of the general characteristics

Univariate analysis was used to compare two groups according to age, gender, marital status, educational level, monthly income and other factors. The results showed that age, residential area, educational level, occupation, monthly income, whether to receive the minimum living allowances, the HIV transmission route and living status had significant effects on the drop-out from ART (*P* < 0.05) Tables 1, 2, 3, 4.

**Table 1:** Comparison of general characteristics between group retained in care and drop out group

Item	therapy status during investigation		*χ2*	*P*
	Drop out group (*n* = 252)	Group retained in care (*n* = 187)		
Gender			0.78	0.38
Male	189 (56.3)	147 (43.8)		
Female	63 (61.2)	40 (38.8)		
Age			11.37	0.04
≤ 24	24 (61.5)	15 (38.5)		
25–34	58 (47.5)	64 (52.5)		
35–44	97 (66.4)	49 (33.6)		
45–54	47 (58.0)	34 (42.0)		
55–64	19 (54.3)	16 (35.7)		
≥ 65	7 (43.8)	9 (56.3)		
Nationality			0.97	0.33
Han nationality	215 (58.4)	153 (41.6)		
Ethnic minority	37 (52.1)	34 (47.9)		
Residential area			39.16	0.00
Rural areas	140 (74.5)	48 (25.5)		
Town	112 (44.6)	139 (55.4)		
Household register			1.85	0.60
Local county	119 (58.6)	84 (41.4)		
Other county of the city	20 (47.6)	22 (52.4)		
Other city of the province	39 (59.1)	27 (40.9)		
Other province	74 (57.8)	54 (42.2)		
Marital status			1.08	0.78
Single	88 (54.3)	74 (45.7)		
Married	71 (60.2)	47 (39.8)		
Divorced	86 (58.5)	61 (41.5)		
Bereaved wife or husband	7 (58.3)	5 (41.7)		
Educational level			31.79	0.00
Illiteracy	10 (90.9)	1 (9.1)		
Primary school	55 (67.9)	26 (32.1)		
Junior high school	111 (66.5)	56 (33.5)		
High school	34 (45.3)	41 (54.7)		
College or above	42 (40.0)	63 (60.0)		
Occupation			57.99	0.00
Farmer	112 (78.9)	30 (21.1)		
Housework and unemployment	65 (56.0)	51 (44.0)		
Service	27 (39.7)	41 (60.3)		
Worker	11 (37.9)	18 (62.1)		
Cadre staff	7 (33.3)	14 (66.7)		
Retiree	1 (8.3)	11 (91.7)		
Student	12 (66.7)	6 (33.3)		
Migrant workers	9 (52.9)	8 (47.1)		
Other	8 (50.0)	8 (50.0)		
Monthly income			33.81	0.00
< 500	84 (75.0)	28 (25.0)		
500–1000	36 (66.7)	18 (33.3)		
1000–2000	35 (36.5)	61 (63.5)		
2000–3000	50 (54.3)	42 (45.7)		
≥ 3000	47 (55.3)	38 (44.7)		
Minimum living allowances			17.50	0.00
Yes	62 (78.5)	17 (21.5)		
No	190 (52.8)	170 (47.2)		
HIV transmission route			43.15	0.00
Injection drug abuse	76 (83.5)	15 (16.5)		
Male to male transmission	43 (40.2)	64 (59.8)		
Heterosexual transmission	133 (55.2)	108 (44.8)		
Living status			10.88	0.01
Live alone	111 (58.4)	79 (41.6)		
Usually live with strangers	20 (83.3)	4 (16.7)		
Live with classmates/colleagues/friends	27 (65.9)	14 (34.1)		
Live with family	94 (51.1)	90 (48.9)		
Recent CD4 counts			0.65	0.723
< 350	126 (59.2%)	87 (40.8%)		
350~	47 (57.3%)	35 (42.7%)		
500~	79 (54.9%)	65 (45.1%)

**Table 2:** A comparative analysis of ART for group retained in care and drop out group

item	therapy status during investigation		*χ2*	*P*
	drop out group (*n* = 252)	group retained in care (*n* = 187)		
First Result Notification institution			6.26	0.28
Centers for Disease Control and Prevention (CDC)	179 (61.5)	112 (38.5)		
Methadone maintenance sites	3 (50.0)	3 (50.0)		
antiretroviral therapy institution	3 (50.0)	3 (50.0)		
Non-governmental Organizations	8 (47.1)	9 (52.9)		
General Hospital	52 (50.5)	51 (49.5)		
Other	7 (43.8)	9 (56.3)		
Referral methods by follow-up institution			100.44	0.00
Carry the card to go by oneself	196 (77.8)	56 (22.2)		
Doctors escort	56 (29.9)	131 (70.1)		
ART information provided by follow-up institutions			14.33	0.00
Yes	222 (54.8)	183 (45.2)		
No	30 (88.2)	4 (11.8)

**Table 3:** Logistic regression analysis about the influencing factors of dropout

item	category	β	S.E	*Waldx*^*2*^	*P*	OR(95%的CI)
Age	≤24				0.465	1.000
	25–34	−0.979	1.001	0.957	0.328	0.376 (0.053~2.673)
	35–44	0.041	0.923	0.002	0.964	1.042 (0.171~6.360)
	45–54	0.029	0.890	0.001	0.974	1.030 (0.180~5.897)
	55–64	−0.269	0.918	0.086	0.769	0.764 (0.126~4.623)
	≥65	−0.400	0.971	0.170	0.681	0.670 (0.100~4.499)
Residential area	Rural areas					1.000
	Town	0.808	0.328	6.051	0.014	0.446 (0.234~0.849)
Education	Illiteracy				0.282	1.000
	Primary school	−2.356	1.323	3.197	0.074	0.094 (0.007~1.256)
	Middle school	−0.852	0.587	2.105	0.147	0.427 (0.135~1.348)
	High school	−0.584	0.503	1.347	0.246	0.558 (0.208~1.495)
	College or above	−0.091	0.478	0.036	0.849	0.913 (0.358~2.331)
Occupation	Farmer				0.248	1.000
	Housework and unemployment	−2.752	1.339	4.227	0.040	0.064 (0.005~0.879)
	Service	−2.022	1.327	2.322	0.128	0.132 (0.010~1.784)
	Worker	−1.699	1.340	1.608	0.205	0.183 (0.013~2.527)
	Cadre staff	−1.345	1.379	0.951	0.330	0.261 (0.017~3.889)
	Retiree	−2.220	1.413	2.470	0.116	0.109 (0.007~1.731)
	Student	−1.862	1.452	1.645	0.200	0.155 (0.009~2.674)
	Migrant workers	−2.322	1.479	2.464	0.117	0.098 (0.005~1.781)
	Other	−2.005	1.433	1.960	0.162	0.135 (0.008~2.231)
Monthly income	< 500				0.016	1.000
	500–1000	0.767	0.505	2.307	0.129	2.153 (0.800~5.795)
	1000–2000	0.522	0.548	0.908	0.341	1.686 (0.576~4.939)
	2000–3000	1.648	0.509	10.490	0.001	5.199 (1.917~14.097)
	≥3000	0.478	0.433	1.220	0.269	1.613 (0.691~3.769)
Minimum living allowances	Yes					1.000
	No	−1.276	0.404	9.988	0.002	0.279 (0.127~0.616)
HIV transmission route	Injecting drug abuse				0.000	1.000
	Male to male transmission	2.317	1.196	3.750	0.053	19.007 (1.869~193.312)
	Heterosexual transmission	1.390	1.235	1.266	0.261	4.014 (0.357~45.198)
Living status	Live alone				0.053	1.000
	Usually live with strangers	−0.665	0.304	4.796	0.029	0.514 (0.283~0.932)
	Live with classmates/colleagues/friends	−1.449	0.749	3.742	0.053	0.235 (0.054~1.019)
	Live with family	−0.820	0.499	2.702	0.100	0.440 (0.166~1.171)
Referral methods by follow-up institution	Carry the card to go by oneself					1.000
	Doctors escort	1.910	0.279	46.931	0.000	0.148 (0.086~0.256)

**Table 4:** Reasons that HIV-infected persons in dropout of ART

Reason	The number of response	The rates of response(%)
side effects are too serious to tolerate	87	34.5
to persist in taking medicine regularly is difficult	72	28.6
medication interruption due to incarcerated	67	26.6
therapy information is asynchronous due to the change of current address	60	23.8
not necessary to continue taking medicine for better health	39	15.5
consider the therapy is ineffective	25	9.9
family members do not support therapy	14	5.6

### Univariate factor analysis of antiretroviral therapy services

Through univariate factor analysis of antiretroviral therapy services provided by HIV/AIDS prevention and control institutions. It was found that referral methods provided by follow-up institutions, whether ART information provided by follow-up institutions were the influencing factors of dropping out from ART (*P* < 0.05).

### Multivariate logistic regression analysis

According to the results of univariate analysis and the inclusion criteria of *P* < 0.05, age, residential area, education level, occupation, monthly income, whether to receive minimum living allowances, HIV transmission route, living status, referral methods provided by follow-up institutions and whether to provide ART information were taken as independent variables. Whether HIV-infected patients dropped out from ART or retain in care as dependent variables (drop out = 0, retain in care = 1), with multivariate Logistic regression analysis. The results showed that HIV-infected patients living in rural areas were the protective factors for the drop out from ART compared with those living in cities or towns (*OR* = 0.446,95%CI:0.234~0.849). HIV-infected patients who has monthly income between 500 to 1000 CNY compared with below 500 CNY (*OR* = 2.153,95%CI:0.800~5.795),more than 3000 CNY compared with 2000–3000 CNY (*OR* = 1.613,95%CI:0.691~3.769) were the risk factors for drop out. Not receiving minimum living allowances compared with who has minimum living allowances (*OR* = 0.279,95%CI:0.127~0.616) was the protective factor for drop out. Patients carrying cards on their own was protective factor for drop out compared with follow-up institutions offered referral method to therapy institution (*OR* = 0.148,95%CI:0.086~0.256).

### Reasons for drop-out from antiretroviral therapy in HIV-infected patients

The top three reasons of drop-out from antiretroviral therapy in HIV-infected patients were: serious side effects, need to persist in taking medicine regularly, medication interruption due to incarcerated.

## Discussion

Since China implemented the policy of “Four Frees and One Care”, the drop-out from ART has been an important issue affecting the therapeutic effect [7]. “Four Frees and One Care”, began in 2003, means: (a) free ART for all AIDS patients in financial difficulty, (b) free schooling for AIDS orphans and children of AIDS patients, (c) free counseling and prevention measures to prevent mother-to -child transmission for HIV-infected pregnant women, and (d) free HIV antibody testing and counseling, provided by the Chinese Center for Disease Control and Prevention.“One Care”means providing cares to AIDS patients and their families. Drop out or not is an important index to measure the patient’s therapy status and the success of therapy. Studies shown that young, male, single or divorced [8], illiterate [9], the proportion of dropping out from therapy is higher. Our study did not show that male more likely to drop out. The higher the level of education is, the more difficult it is to drop out, which was consistent with the results of Mison Dahhab [10].

Our study found that injection drug abusers prone to drop out, which was consistent with previous studies on the impact of injecting drug use on drop out [11]. Drug addicts usually managed by different institutions and departments, such as detoxification centers, prisons, methadone clinics, community antiretroviral therapy services institutions and so on. Work coordination among institutions should be strengthened to ensure that patients can receive sustained antiretroviral therapy in all institutions.

Multivariate analysis shows that residential area is the factor influencing the drop-out from ART. The HIV patients who living in rural areas easier to drop-out from ART than patients live in urban areas. On the one hand, because most antiretroviral therapy institutions setting in cities or towns, the availability of antiviral drugs for HIV-infected patients in rural areas was not high due to long travel distances from rural area to therapy institution and need pay out-of-pocket transportation expenses. Foreign studies also showed that too long distance to get drugs in hospitals was one of the factors affecting the maintenance therapy of patients [12]. It is suggested that more antiretroviral therapy points added within a reasonable distance. Meanwhile, we suggest that ART medicine could delivered to HIV infected patients under the premise of strict confidentiality of personal privacy. On the other hand, some patients changed their Kunming mobile phone number, which made the original therapy institutions unable to contact them successfully. Therefore, doctors should inform patients of referral information during therapy. In addition, they should leave contact information as many as possible which not easy to change such as QQ or WeChat. The medicine users need actively cooperate with doctors.

Multivariate analysis shows that the monthly income level and whether to receive a minimum living allowance are also important factors affecting the drop-out from ART. Because HIV-infection patients who have lower monthly income could receive minimum living allowance from government,so this kind of people more easier drop out of ART due to their lower financial level. Since the introduction of the ‘four frees and one care’ policy in 2003, our government has effectively provided more and more welfare policies for antiretroviral therapy. However, parts of examination fees and transportation costs need to be paid by individuals. This may be the reason for low-income HIV-infected patients drop out from ART. It’s indicated that reducing the cost of examination for patients with financial difficulties deserves attention.

Our study includes a survey of antiretroviral therapy related medical services in the questionnaire. It is found that the mode of referral provided by follow-up agencies is an important factor affecting the drop-out from antiretroviral therapy. Accompanied referral can greatly reduce the drop out rate of antiretroviral therapy, which is rarely mentioned in other studies. By establishing a bridge between follow-up agencies and antiretroviral therapy points, the time from receiving their HIV diagnosis to timely therapy can be significantly shortened, the therapy of HIV-infected patients can be promoted, and the drop-out from ART can be reduced [13]. It is suggested that further study should be conducted on the role of accompany referral in reducing the drop out rate.

Our study found that HIV-infected patients consider that the main reason of the drop out was drug side effects. Others’ Studies have shown that the discomfort caused by side effects is one of the important reason of HIV-infected patients difficult to maintain of ART therapy [14]. Therefore, health workers are required to carry out effective compliance education before antiretroviral therapy, increase times of early follow-up visits. Medicine side effects should be dealt with in time, therapy plans should be adjusted as appropriate.

## Limitations

Our study has limitations. It is a cross-sectional study with small sample size. And all the influencing factors are not included in the analysis, such as: opportunistic infections before therapy or not, virology failure and other factors, which may cause bias. Therefore, it is necessary to track the drop-out from therapy of HIV-infected patients in the future’s study.

### Conclusion

This study identified a number of factors associated with drop out from ART. Based on our findings,appropriate interventions should be introduced decrease drop-out. We should pay attention to HIV-infected patients who is injecting drug user, low educational level or low economic level. Government provide more financial supports and strengthen AIDS-related medical services, so as to effectively improve the therapy retention rate and reduce the drop out rate of ART.

## Data Availability

The data used in this study are available from the corresponding author on reasonable request.

## Abbreviations

AIDS: Acquired Immune Deficiency Syndrome
ART: Antiretroviral therapy
CI: Confidence interval
HIV: Human immunodeficiency virus
OR: Odds ratio
UNAIDS: United Nations Programme on HIV/AIDS
